# Biogeochemical consequences of a changing Arctic shelf seafloor ecosystem

**DOI:** 10.1007/s13280-021-01638-3

**Published:** 2021-10-09

**Authors:** Christian März, Felipe S. Freitas, Johan C. Faust, Jasmin A. Godbold, Sian F. Henley, Allyson C. Tessin, Geoffrey D. Abbott, Ruth Airs, Sandra Arndt, David K. A. Barnes, Laura J. Grange, Neil D. Gray, Ian M. Head, Katharine R. Hendry, Robert G. Hilton, Adam J. Reed, Saskia Rühl, Martin Solan, Terri A. Souster, Mark A. Stevenson, Karen Tait, James Ward, Stephen Widdicombe

**Affiliations:** 1 School of Earth and Environment, University of Leeds, Leeds, LS2 9JT UK; 2 School of Earth Sciences, University of Bristol, Wills Memorial Building, Queens Road, Bristol, BS8 1QE UK; 3 MARUM—Center for Marine Environmental Sciences, University of Bremen, Leobener Strasse 8, 28359 Bremen, Germany; 4 Ocean and Earth Science, National Oceanography Centre Southampton, University of Southampton, Waterfront Campus, European Way, Southampton, SO14 3ZH UK; 5 School of GeoSciences, University of Edinburgh, James Hutton Road, Edinburgh, EH9 3FE UK; 6 Department of Geology, Kent State University, 221 McGilvrey Hall, 325 S. Lincoln St., Kent, OH 44242 USA; 7 School of Natural and Environmental Sciences, Newcastle University, Newcastle upon Tyne, NE1 7RU UK; 8 Plymouth Marine Laboratory, Prospect Place, Plymouth, PL1 3DH UK; 9 Department of Geosciences, Environment and Society, Université libre de Bruxelles, Brussels, Av. F. Roosevelt 50, CP160/02, 1050 Brussels, Belgium; 10 British Antarctic Survey, UKRI, High Cross, Maddingley Rd, Cambridge, CB3 0ET UK; 11 School of Ocean Sciences, Bangor University, Bangor, Gwynedd, LL57 2DG North Wales UK; 12 Department of Geography, Durham University, Lower Mountjoy, South Rd, Durham, DH1 3LE USA; 13 Helmholtz Zentrum Hereon, Max-Planck-Straße 1, 21502 Geesthacht, Germany; 14 Department of Biosciences, Fisheries and Economics, UIT, Tromsø, Norway

**Keywords:** Arctic Ocean, Biogeochemistry, Carbon, Ecology, Nutrients, Trawling

## Abstract

Unprecedented and dramatic transformations are occurring in the Arctic in response to climate change, but academic, public, and political discourse has disproportionately focussed on the most visible and direct aspects of change, including sea ice melt, permafrost thaw, the fate of charismatic megafauna, and the expansion of fisheries. Such narratives disregard the importance of less visible and indirect processes and, in particular, miss the substantive contribution of the shelf seafloor in regulating nutrients and sequestering carbon. Here, we summarise the biogeochemical functioning of the Arctic shelf seafloor before considering how climate change and regional adjustments to human activities may alter its biogeochemical and ecological dynamics, including ecosystem function, carbon burial, or nutrient recycling. We highlight the importance of the Arctic benthic system in mitigating climatic and anthropogenic change and, with a focus on the Barents Sea, offer some observations and our perspectives on future management and policy.

## Introduction

**Fig. 1:**
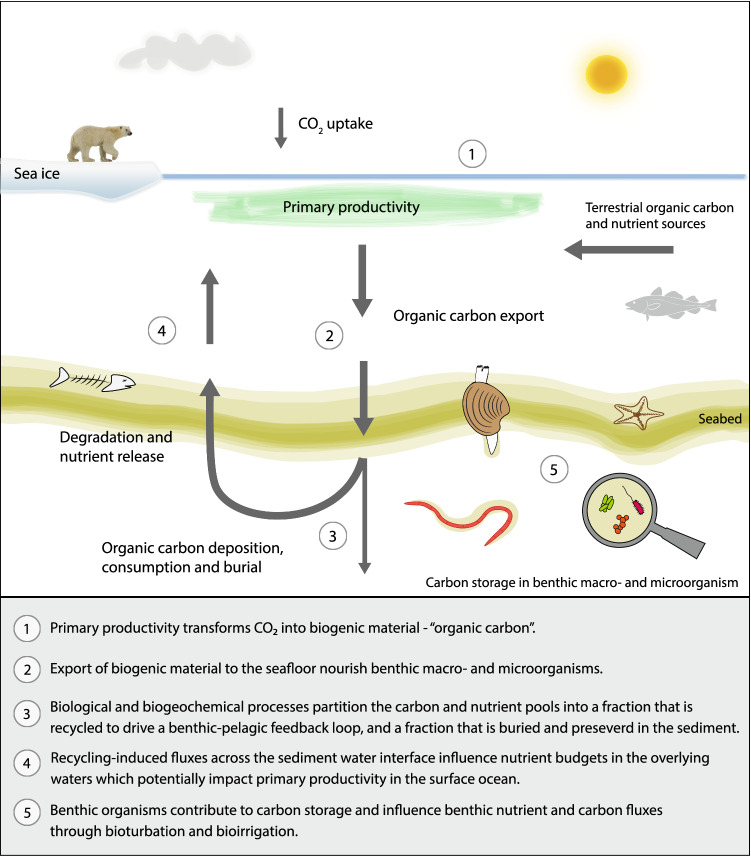
Schematic illustration of ecological and biogeochemical parameters in Arctic Ocean shelf seas, with a focus on processes at the seafloor

The Arctic Ocean seafloor hosts a diverse and productive benthic ecosystem that is a crucial component of an intimately coupled benthic–pelagic system (Fig. 1; Piepenburg 2005). Benthic organisms modulate sequestration, transformation, and storage of bio-essential nutrients and carbon across the Arctic shelf seas (Morata et al. 2020). A significant proportion of organic matter (OM) from marine, terrestrial, or sea ice sources is further recycled via microbially mediated processes that are coupled to the activities of benthic meio-, macro-, and megafauna (e.g. via bioturbation, bioirrigation; Piepenburg et al. 1995; Renaud et al. 2008). These biological and biogeochemical processes partition the carbon and nutrient pools into a fraction that is recycled to drive a benthic–pelagic feedback loop, and a fraction that is buried in the sediment. On the shallow Arctic shelf, the feedback with water column processes (via physical mixing and primary productivity) is more pronounced than in the deep ocean and plays a crucial role for benthic–pelagic coupling and ecosystem productivity; the latter could then contribute to the long-term removal of carbon from the ocean–atmosphere system. Key uncertainties exist, however, in how changing sea ice dynamics (e.g. thickness, extent, inter-annual variability) will alter existing biological community composition and structure, biogeochemical processes, and associated ecosystem functioning. Understanding how these responses are manifest in the benthic environment, both directly and indirectly, is crucial to understanding the Arctic ecosystem as a whole and its importance at the larger scale (Macdonald et al. 2015).

One frequently debated proposition on Arctic change is that longer and more extensive open water conditions, especially across Arctic shelves, could lead to prolonged growing seasons and enhanced CO_2_ uptake by biomass (Arrigo and Van Dijken 2015; Slagstad et al. 2015). Eventually, this could result in a negative feedback on the CO_2_-induced greenhouse effect in the Arctic as more carbon is sequestered into the sediment. However, modelling the response of the Arctic Ocean carbon and nutrient cycles to reduced sea ice and its associated, and partly counteracting, effects (deeper light penetration, longer growth seasons, increased water column stratification, ocean acidification, warming), is difficult—partly due to an incomplete mechanistic understanding of the changing Arctic Ocean seafloor. It is currently unclear which fraction of carbon and nutrients will be metabolised and transformed at the seafloor, which interactions between microbial and macro-benthic activity dominate these transformations, and what the effects are on ecosystem structure and functioning. Seafloor recycling likely plays a significant role for the whole Arctic Ocean, with associated societal feedbacks on fisheries and other marine resources, highlighting the critical importance of understanding and quantifying biogeochemical processes at the Arctic seafloor. The carbon storage potential of marine sediments in particular has only recently been recognised and evaluated (Luisetti et al. 2020). Aspects to consider here are the reliable knowledge of carbon contents, the vulnerability of this carbon store, and its assignment to specific nations. These questions will be relevant for designing governance frameworks on sediment carbon storage, but there is little empirical support to the assumed carbon inventory. Although sophisticated, multi-component diagenetic models now exist, most regional- to global-scale biogeochemical and Earth system models do not resolve the complexity of the seafloor environment. Moreover, models tend to neglect or simplify biogeochemical processes by using a limited number of parameters in the sediment and, in so doing, misrepresent organism–sediment interactions and benthic–pelagic coupling (Lessin et al. 2018; LaRowe et al. 2020).

With the recognition that the Arctic is undergoing transformative, and possibly irreversible, changes come a need to re-evaluate how external forcing could change the fundamentals of the system. For context, we describe the role of the Arctic Ocean seafloor in carbon and nutrient cycling, OM burial, and ecological function, provide context of how this role might change in the future, use a reaction-transport model to estimate possible changes to carbon and nutrient cycling in the Barents Sea, and give perspectives on human activities and management.

## Biogeochemical functioning of the Arctic shelf seafloor: Recycling versus storage

Fundamentally, benthic recycling of carbon and nutrients is driven by the supply of biogenic material to the seafloor, and its subsequent degradation and dissolution (Fig. 1; e.g. Middelburg 2019). Rates of seafloor recycling are enhanced by intense activity of macro- and microorganisms, such as faunal feeding, sediment mixing, and microbial degradation. Recycling-induced fluxes across the sediment–water interface influence nutrient budgets in the overlying waters (e.g. Bourgeois et al. 2017), which, in turn, can impact primary production in the surface ocean. Any carbon that escapes benthic recycling gets preserved below the seafloor, and this carbon burial is crucial for transferring atmospheric CO_2_ to a long-term sediment store. It is this balance between benthic recycling and storage of carbon and nutrients that is likely to change in the future Arctic shelf seas.

**Fig. 2:**
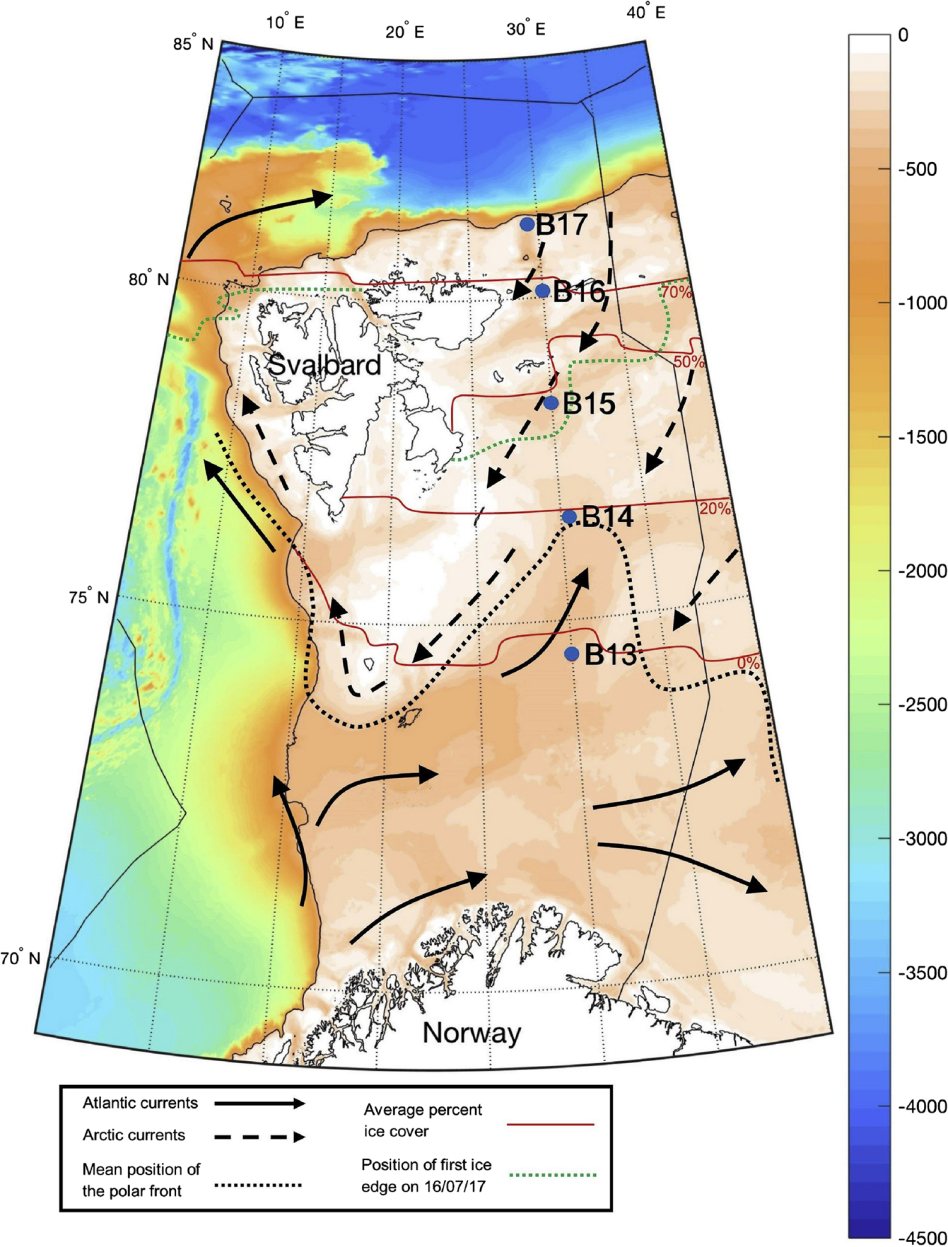
Location of Barents Sea shelf stations B13–B17 sampled in July 2017. Bathymetric depth chart indicating metres below sea level (m.b.s.l.). Depths of sampling were 359 m at B13 (74° 29.998 N, 30° 00.009 E), 293 m at B14 (76° 30.055 N, 30°30.241E), 317 m at B15 (78° 15.100 N, 30° 00.540 E), 283 m at B16 (80° 07.154 N, 30° 04.069 E), and 340 m at B17 (81° 16.765 N, 30° 19.496 E). From Stevenson and Abbott (2019)

In terms of carbon and nutrient cycling, Arctic shelf seas (e.g. the Barents Sea) are special because (i) they are often highly productive, with significant atmospheric CO_2_ uptake (Arrigo and van Dijken 2015); (ii) their shallow waters allow for a fast transfer of OM to the seafloor; and (iii) strong seasonality and cold temperatures allow for efficient, pulsed carbon transfer to the seafloor (Wassmann et al. 2006a, b; DeVries and Weber 2017). Once at the seafloor, the fate of carbon and nutrients depends on the quality and quantity of exported OM (Morata and Renaud 2008; Stevenson et al. 2020), the stability of sedimentary OM and nutrients linked to reactive iron phases in the upper sediments (Faust et al. 2020, 2021), and the composition and process rates of benthic biota (McTigue et al. 2016; Solan et al. 2020a, b). For the Barents Sea, recent models (Freitas et al. 2020) suggest that benthic recycling of nutrients from sediments to overlying waters is mainly controlled by OM reactivity, and therefore, its source, age, and total amount (Fig. 2). In addition, this study shows the magnitude of nutrient fluxes to be somewhat independent from sea ice extent and, instead, to be mostly impacted by the (physico-chemical) structure of the overlying waters (Freitas et al. 2020). With the pronounced changes in Arctic Ocean ecosystems (e.g. changes in sea ice, water masses, phytoplankton species) that are projected to intensify in the coming decades (e.g. Årthun et al. 2012; Smedsrud et al. 2013; Oziel et al. 2017, 2020; Lewis et al. 2020), the trajectory of carbon and nutrient recycling at the seafloor is uncertain.

## Altered system expressions and dynamics

Available evidence suggests that conditions across the Barents Sea, and other Arctic inflow shelves, will become more akin to those of sub-Arctic seas. Warming is predicted to promote Barents Sea ‘Atlantification’ and Chukchi Sea ‘Pacification’ whereby warmer, saltier, and nutrient-richer waters routinely expand further north, often leading to higher primary productivity (Barton et al. 2018; Lind et al. 2018). If sea ice reduction is paralleled by enhanced vertical mixing (Lind et al. 2018; Randelhoff et al. 2020), phytoplankton growing seasons are extended. Enhanced mixing and bloom duration could shift nutrient demands (Downes et al. 2021), with knock-on effects on carbon export. It should be noted, however, that due to the environmental complexities, there is significant uncertainty in any prediction of Arctic Ocean primary productivity (Vancoppenolle et al. 2013). In addition, thawing permafrost is now prevalent around the Arctic Ocean (in particular in Siberia) which, combined with higher river runoff, will deliver more carbon and nutrients to the Arctic shelves (e.g. Bröder et al. 2018; Terhaar et al. 2021). These changes in the status quo will likely alter pathways of carbon delivery to the seafloor and, in turn, the amount of carbon preserved within sediments. Further, changes in the composition and behaviour of the benthic community will affect the fate of both organic and inorganic carbon accumulation at the seafloor. While there is a basic understanding of current factors affecting Arctic seafloor biogeochemistry, some controls on OM burial play out over thousands of years (e.g. Faust et al. 2021). It is unknown if ongoing/future climate change may perturb these processes, either by modifying carbon inputs and/or the microbial communities and degradation pathways below the seafloor (Brüchert et al. 2018). In addition, while the burial of zoobenthic carbon may be more strongly affected by ecosystem change (i.e. the dominant benthic fauna), no clear link between this carbon pool and the position of the sea ice margin was found in the Barents Sea (Souster et al. 2020). This may be partly due to the limited number of habitats studied, or the numerous and complex interactions along the process chain from sea ice cover and carbon export to dynamic ecosystem responses. At similar water depths around Antarctica, across-habitat studies have suggested that maximum burial may occur in habitat interface zones, e.g. where basins meet glacial moraines (Barnes and Sands 2017).

Intimately linked to OM deposition at the seafloor is the cycling of nutrients. Benthic nutrient recycling rates and fluxes are highly sensitive to the impacts of primary production and OM export changes (e.g. Freitas et al. 2021). Extension of the phytoplankton growing season in the Barents Sea carries with it the potential to increase total primary production if sufficient nutrients are available (e.g. Henley et al. 2020; Lewis et al. 2020). Should this occur, and translate into greater export of ‘fresh’ OM, it could lead to higher benthic nutrient fluxes, although any effect is unlikely to be universally expressed due to strong regional differences (e.g. Downes et al. 2020; Oziel et al. 2020). Indeed, the highly seasonal, often short-term, and highly regional benthic–pelagic dynamics on Arctic shelves go some way in explaining why an often assumed link between sea ice cover and benthic nutrient fluxes is not always found (Freitas et al. 2020). This contrasts with sediment carbon dynamics, with seasonally ice-covered parts of the Barents Sea exhibiting lower organic carbon contents, but higher organic carbon burial rates (Faust et al. 2020) and higher abundances of benthic fauna (Souster et al. 2020). On Arctic shelves and margins currently more permanently ice covered (e.g. Yermak Plateau), changes in primary production and OM delivery to the seafloor can lead to comparatively greater changes in benthic nutrient fluxes as compared to the low background values (Tessin et al. 2020).

While no systematic relationship between benthic nutrient fluxes and sea ice cover was found in the Barents Sea, there is a significant link with water mass distributions and ‘Atlantification’. Benthic nutrient fluxes in summer 2017 were higher at stations dominated by Atlantic water (B13, B14, B17; Fig. 2) than at those dominated by Arctic water (B15, B16; Fig. 2) (Freitas et al 2020). If ‘Atlantification’ continues, benthic nutrient fluxes are likely to increase across the region, irrespective of superimposed seasonal and spatial variability. However, patterns of response will depend on the relative importance of, and interactions between, increased bottom water temperatures, changes in primary production and phytoplankton communities, and OM delivery to the seafloor. And since the benthic efflux depends on fixation of nutrients in deposited organic biomass, a net addition to benthic nutrient effluxes will only occur if the Barents Sea system as a whole receives increased external nutrients, for example, through Atlantic water (Oziel et al. 2017) or by increased input (and degradation) of terrestrial OM (Terhaar et al. 2019, 2021).

## Estimating future organic carbon burial and benthic nutrient cycling using a reaction-transport model

**Fig. 3:**
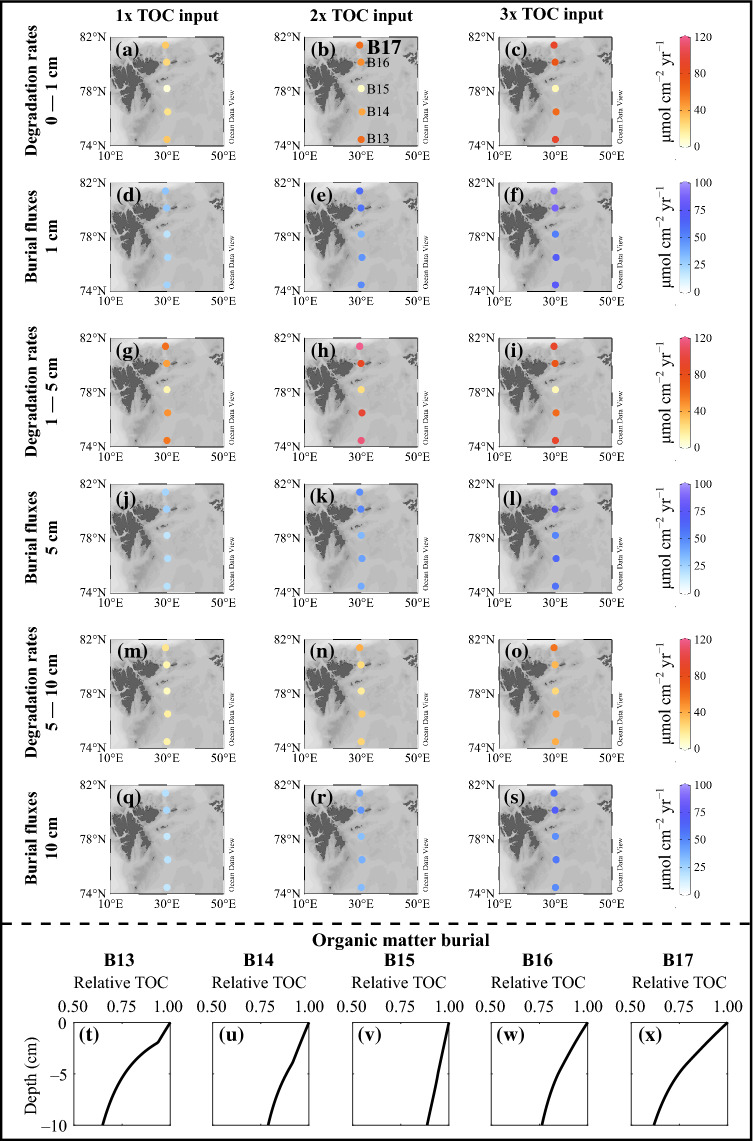
Changes in degradation and burial rates of total organic carbon (TOC) following increased OM export to the seafloor at the Barents Sea sites B13–B17. Model adopted from Freitas et al. (2020), with outputs based on data gathered in July 2017. Integrated TOC degradation rates (warm colour bar) are shown for intervals **a**–**c** 0–1 cm, **g**–**i** 1–5 cm, and **m**–**o** 5–10 cm sediment depth. Corresponding TOC burial rates (cold colour bar) are shown at **d**–**f** 1, **j**–**l** 5, and **q**–**s** 10 cm sediment depth. **t**–**x** Relative fraction of TOC burial with increasing burial depth (cm) in response to input at sediment surface

Working from the realistic assumption (for reasons stated above) that reduced sea ice in the Barents Sea may lead to increased OM export to the seafloor, we estimate the impact of this on carbon burial and degradation rates by performing a simple model sensitivity analysis (Fig. 3). We use our baseline model for the Barents Sea shelf (Freitas et al. 2020) that is confounded in biogeochemical data from five key stations across the Polar Front in the summers of 2017–2019 (Fig. 2). Here, we test how relative fluctuations in OM input (1–3 times the baseline values; expressed as total organic carbon, TOC) to the seafloor translate into absolute and relative changes in burial and degradation rates. While an increase in OM export to the seafloor from primary productivity will impact OM degradation pathways, the impact on long-term sediment carbon burial will be minor, as phytoplankton OM is quickly degraded at the seafloor (Fig. 3). However, we also observe that the fraction of carbon preserved at depth is highest at stations B15 and B16 (just north of the Polar Front), for poorly known reasons but presumably related to the dominance of Arctic water and/or seasonal sea ice at those stations. How much of the carbon delivered into shelf seas by permafrost thaw, coastal erosion, and major river systems is degraded before burial is debated (e.g. Tank et al. 2012; Bröder et al. 2018, 2019; Brüchert et al. 2018;) and further complicated by lateral OM transport along the shelf (Stevenson et al. 2020). Nevertheless, terrestrial processes will likely exert a major control on OM quality/quantity by delivering less degradable OM to Barents Sea sediments (Freitas et al. 2020). Impacts of higher OM fluxes on zoobenthic carbon standing stocks are poorly studied in the Arctic but, in West Antarctic shelf seas, extended phytoplankton blooms promoted by sea ice loss have led to a doubling of zoobenthic carbon standing stock (Barnes 2015, 2017). It is tempting, therefore, to suggest that a similar development might occur on Arctic shelves.

**Fig. 4:**
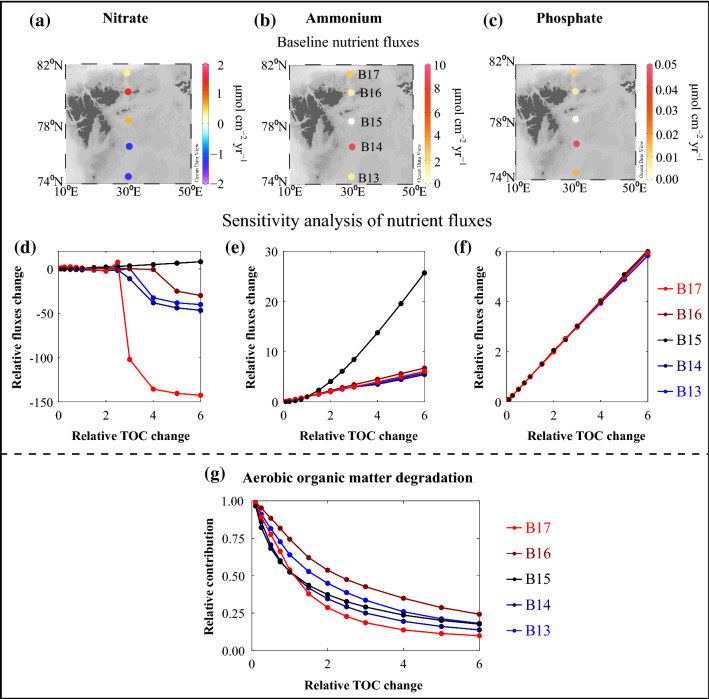
Changes in biogeochemical parameters following increases in OM export to the seafloor at the Barents Sea sites B13–B17. Model adopted from Freitas et al. (2020), with outputs based on data gathered in July 2017. Top row: baseline nutrient fluxes of **a** nitrate, **b** ammonium, and **c** phosphate. Note the different scales in the colour bar and direction of fluxes: cold colours denote fluxes into sediments; warm colours denote fluxes out of the sediment. Middle row: changes in nutrient fluxes of **d** nitrate, **e** ammonium, and **f** phosphate relative to increased OM input. Note different scales in relative flux changes (*y*-axis) due to nutrient-specific response to OM input and transformation at the seafloor: **d** nitrate fluxes become negative (i.e. sediments acting as nitrate sink rather than source), while **e** ammonium and **f** phosphate fluxes increase. Line colours **d**–**g** denote reference sites in the Barents Sea. Bottom row: **g** changes in relative contribution of aerobic (presence of oxygen) OM degradation with gradual increase in OM input. Contribution of aerobic OM degradation decreases exponentially with higher OM input, which slows down overall degradation of OM

In a second step, to estimate the impacts of OM export changes on benthic nutrient fluxes (ignoring ecological drivers), we expand a simple model sensitivity analysis used for TOC degradation and burial rates (after Freitas et al. 2020) to calculate benthic nutrient fluxes (nitrate, ammonium, phosphate; Fig. 4). We change the OM content to 0.1–6 times relative to baseline values, keeping all other model parameters unchanged. Our simulation shows that any fluctuation in OM input to the seafloor will result in a concomitant adjustment in nutrient fluxes (Fig. 4), even though the responses are not strictly linear, vary between sites, and are nutrient-specific. Our results also suggest that absolute changes in nutrient fluxes are likely to be more pronounced at sites influenced by Atlantic Water, and that relative increases in OM input will trigger large changes in the way OM is being degraded at and below the seafloor. The relative contribution of aerobic OM degradation will decrease considerably as oxygen will become quickly depleted (Fig. 4), while anaerobic conditions will prevail in the upper end of OM addition scenarios.

It should be noted that changes to ecological factors were ignored in the modelling exercise above, but there is no doubt that environmental and anthropogenic change will also affect the benthic ecosystem. A faunal separation occurs between northern (Arctic) and southern (Atlantic) assemblages at the operational Polar Front (e.g. Jørgensen et al. 2015). The distribution of functionally important species has received some attention (Degen and Faulwetter 2019), but there are few direct measurements of faunal activity or physiological state, and no regional-scale assessments of the faunal mediation of biogeochemistry (Solan et al. 2019). Nevertheless, recent observations in the Barents Sea indicate that spatial and temporal variability in environmental setting will be important in explaining biodiversity and ecological functions at larger scales, more so than localised sea ice changes (Solan et al. 2020a, b; Souster et al. 2020; Oleszczuk et al. 2021). Changes in the quality and quantity of OM reaching the seabed can have significant implications for faunal physiology, behaviour, growth (Reed et al. 2021a), and reproduction (Reed et al. 2021b) and, in turn, biogeochemical cycling (Solan et al. 2020a, b). Overall, however, there is a clear south–north increase in species richness, biomass, and functional diversity of mega- and macro-zoobenthos, but the mixed depth of sediment and bioturbating activity of the community both decline with increasing latitude (Solan et al. 2020a, b; Souster et al. 2020).

## Climate- and human-induced changes

The preservation of carbon within shelf sediments and benthic marine communities is likely to be altered by the expansion of human activities as sea ice retreats, including fishing, shipping, and petroleum exploration. With less challenging sea ice conditions and the northward migration of economically valuable fish stocks (e.g. Atlantic cod *Gadus morhua*, Greenland halibut *Reinhardtuis hippoglossoides*, shrimp *Pandalus borealis*), commercial fisheries follow and start trawling some of the last unfished areas of the global shelf seafloor. Bottom trawling causes re-working and re-suspension of seafloor sediment (Puig et al. 2012; O’Neill and Ivanović 2016), which can lead to erosion and perturbations to benthic biogeochemistry, in particular a loss of sedimentary organic carbon (Paradis et al. 2021). However, in the Barents Sea, reactive OM is quickly degraded and recycled to CO_2_ within the surface sediments (Freitas et al. 2020; Stevenson et al. 2020), even without human intervention. The question then arises as to whether trawling will impact the more stable, deeper, pre-degraded carbon stocks that remain in the sediments. This will depend on various factors, including the depth of trawl penetration (typically 10s of cm) and the overall sediment accumulation rates (~ 4–200 cm/1000 years; Faust et al 2020): Under high sedimentation rates, reactive OM is buried relatively quickly, and re-exposure by trawling would negatively affecting overall carbon burial efficiency. In low sedimentation rate areas, trawling might have less of an effect on long-term carbon storage. Similar considerations can be made for nutrient recycling to the water column by the mechanical disturbance of sediments (Duplisea et al. 2001): if the disturbance reaches anaerobic layers where nutrient concentrations are significantly higher than in the overlying waters, the resulting enhanced nutrient fluxes can fuel additional pelagic primary production (Dounas et al. 2007; van der Velde et al. 2018; Tiano et al. 2021). Finally, the persistence of any trawling-induced disturbance in the Barents Sea would depend on type and frequency of trawling as well as primary productivity and sedimentation rates, but literature-based estimates range from several year to several decades (Buhl-Mortensen et al. 2016; Paradis et al. 2021).

Besides the sediment, polar benthic marine communities also store considerable carbon in the form of biota. Zoobenthic carbon in the Barents Sea is comparable to the highest levels in Antarctic shelf sediments (Souster et al. 2020). Changes in the density, diversity, and composition of mega-benthic communities associated with bottom fishing activity in the Barents Sea have been observed (Buhl-Mortensen et al. 2016) and can significantly affect the biomass and stored carbon of all species (Jørgensen et al. 2016).

## Implications for management and policy

Warming, in combination with increased disturbance of the Arctic shelf seafloor, is already imposing significant changes to carbon and nutrient cycles, as well as ecosystems. Following scientific recommendation, areas with fishing restrictions or closure in the Barents Sea, particularly around Svalbard, were recently expanded by the Norwegian government (Jørgensen et al. 2020). The ecosystem protection afforded by MPA or similar protection status increased the likelihood of safeguarding carbon stocks and the processes that control seafloor carbon sequestration (Atwood et al. 2020; Sala et al. 2021). For example, modifying fishing gears, limiting or preventing seafloor trawling would reduce the physical disturbance that alters community composition and diversity, biogeochemical cycling, and the amount of carbon released back into the water (Duplisea et al. 2001; Dounas et al. 2007; Tiano et al. 2019). However, expansion of fishery exclusion zones in the Barents Sea is based largely on ecological/biodiversity criteria, rather than on the need for protecting carbon stocks (Jørgensen et al. 2020). Recognition of the carbon burial aspect of marine ecosystem services is currently missing in Arctic seas, but is increasingly recognised elsewhere (Atwood et al. 2020; Luisetti et al. 2020; Sala et al. 2021). Biologically rich, vulnerable marine environment hotspots can also be effective carbon sinks, as in the case of the first high seas MPA around the South Orkney Islands, Antarctic Peninsula (Trathan et al. 2014; Barnes et al 2016). Consideration of both nature and its functionality (ecosystem services or nature-based solutions, Solan et al. 2020a, b) provides a stronger and more comprehensive approach compared to a focus on biodiversity alone (e.g. Sala et al. 2021). Societal and scientific pressure has recently resulted in creation of some Very Large Marine-Protected Areas (VLMPAs) but, as Sala et al. (2021) note, this includes few areas within the polar regions. The polar regions have more governance complexity than most Exclusive Economic Zones (EEZs), but they lag behind global MPA creation, even though they could present new opportunities for carbon store protection. For example, 99% of most of Ascension Island’s VLMPA is deeper than 1000 m, but the main carbon pathway to sequestration may occur in the shallowest 1000 m (Barnes et al. 2019). Protection of this shallow seabed safeguards £1–2 million of carbon capture to sequestration at UN shadow price of carbon estimates. There are opportunities in the Arctic to target such shallow carbon burial grounds. It is crucial to learn lessons from rushed MPA designations, since those are often agreed on economically unattractive areas, or implemented with clauses that allow resource exploitation to continue.

Society has to decide the type, rate and level of human activity that is acceptable in Arctic regions, while balancing competing demands and world views, and to agree on equitable ways to resolve conflict and maximise win–win strategies. However, the data needed to support effective marine management within the Arctic are sparse, incomplete or poorly quantified, making planning and more informed decision-making challenging. Even in the better investigated regions such as the Barents Sea, only parts of the carbon pathway (from capture to sequestration) are quantified and—even then—only for some habitats (e.g. muddy glacial troughs; Faust et al. 2020; Freitas et al. 2020; Solan et al. 2020a, b; Souster et al. 2020; Stevenson et al. 2020). When appropriate socio-ecological data do exist, the focus is spatially constrained and in a limited number of areas (Falardeau and Bennett 2020). However, we understand enough to know that vulnerable marine ecosystems on Arctic continental shelves are not necessarily co-located with the main carbon burial environments. The most productive and most heavily fished ecosystems are situated on shoals, around the coasts and above rocky ground, while most organic carbon is likely sequestered in muddy sediments of glacial troughs. We also know that high productivity and throughput of carbon do not necessarily mean high carbon sequestration. The prevailing systems controlling the cycling and storage of carbon in the Arctic seafloor are complex, and there is a general paucity of fully comprehensive datasets. Despite the challenges, it is possible to make considerable progress in identifying the most significant unprotected carbon burial hotspots, allowing for an effective assessment of the landscape of potential threats and the risks and rewards surrounding seafloor protection. Most ecosystems affected by human disturbance can recover when conditions improve, for example, if appropriate conservation measures are enacted and human pressure is managed (Jones and Schmitz 2009). To continue to benefit from seafloor carbon sinks and buy more time against climate change, we contend that MPAs (no bottom fishing) for newly exposed ice-free regions in the Arctic will be beneficial.
